# Deep neural networks show an equivalent and often superior performance to dermatologists in onychomycosis diagnosis: Automatic construction of onychomycosis datasets by region-based convolutional deep neural network

**DOI:** 10.1371/journal.pone.0191493

**Published:** 2018-01-19

**Authors:** Seung Seog Han, Gyeong Hun Park, Woohyung Lim, Myoung Shin Kim, Jung Im Na, Ilwoo Park, Sung Eun Chang

**Affiliations:** 1 I Dermatology, Seoul, Korea; 2 Department of Dermatology, Dongtan Sacred Heart Hospital, Hallym University College of Medicine, Dongtan, Korea; 3 SK Telecom, HMI Tech. Lab, Seoul, Korea; 4 Department of Dermatology, Sanggye Paik Hospital, Inje University College of Medicine, Seoul, Korea; 5 Department of Dermatology, Seoul National University College of Medicine, Seoul, Korea; 6 Department of Radiology, Chonnam National University Medical School and Hospital, Gwangju, Korea; 7 Department of Dermatology, Asan Medical Center, Ulsan University College of Medicine, Seoul, Korea; Tokai University, JAPAN

## Abstract

Although there have been reports of the successful diagnosis of skin disorders using deep learning, unrealistically large clinical image datasets are required for artificial intelligence (AI) training. We created datasets of standardized nail images using a region-based convolutional neural network (R-CNN) trained to distinguish the nail from the background.

We used R-CNN to generate training datasets of 49,567 images, which we then used to fine-tune the ResNet-152 and VGG-19 models. The validation datasets comprised 100 and 194 images from Inje University (B1 and B2 datasets, respectively), 125 images from Hallym University (C dataset), and 939 images from Seoul National University (D dataset).

The AI (ensemble model; ResNet-152 + VGG-19 + feedforward neural networks) results showed test sensitivity/specificity/ area under the curve values of (96.0 / 94.7 / 0.98), (82.7 / 96.7 / 0.95), (92.3 / 79.3 / 0.93), (87.7 / 69.3 / 0.82) for the B1, B2, C, and D datasets.

With a combination of the B1 and C datasets, the AI Youden index was significantly (*p* = 0.01) higher than that of 42 dermatologists doing the same assessment manually. For B1+C and B2+ D dataset combinations, almost none of the dermatologists performed as well as the AI. By training with a dataset comprising 49,567 images, we achieved a diagnostic accuracy for onychomycosis using deep learning that was superior to that of most of the dermatologists who participated in this study.

## Introduction

Although convolutional neural networks (CNNs), which are based on a deep-learning algorithm, have diagnosed diabetic retinopathy and skin cancer with an accuracy that is comparable to specialist clinicians, a large number of clinical photographs are required to train the CNNs.[1, 2] There are several CNN models available, such as AlexNet, VGG, GoogLeNet, Inception and ResNet. AlexNet won the 2012 ImageNet Large-Scale Visual Recognition Challenge (ILSVRC) and at that time represented a notable improvement over the conventional hand-crafted feature extraction algorithms. Conventional methods had never really performed well and led to the use of the term ‘Moravec’s Paradox’, i.e. it is difficult for the computer to distinguish simple subjects such as cats and dogs even though this is easily done even by children. Since 2012, all models that have won the ILSVRC have been based on the deep learning algorithm. Visual geometry group (VGG) is a CNN model developed by the University of Oxford that showed that deeper networks can give better results.[3] Microsoft ResNet-152 is an extremely deep 152-layer CNN model that can learn features at various levels of abstraction to boost performance.[4, 5] ResNet-152 recently won the 2015 ILSVRC with an error rate of 3.6%, outperforming the person who participated in their experiment.[5] The ResNet-152 architecture surpass AlexNet, VGG-19, and other old architectures by a significant margin of at least 7% in one-crop Top-1 accuracy.[6]

Region-based CNN (R-CNN) is a type of CNN that can detect the location of lesions of interest by distinguishing them from the background.[7] While CNN is used to determine whether or not the image is matched to the desired object, beyond determining an object of the CNN, the R-CNN can locate the desired object within the image. R-CNN is a combination of region proposals with CNNs, and various CNN models can be applied with region proposal algorithms.

CNN systems become more accurate as the data volumes get larger. Hence, the biggest obstacle to accuracy using deep learning algorithm in the medical field today is the lack of datasets. Our current study was designed to investigate whether AI (CNN) can achieve a performance above that of a clinical specialist by resolving dataset deficiencies with the help of another AI (R-CNN). We selected onychomycosis among the possible skin disorders with which to test our system because 1) it is a wide-spreading disease, 2) unlike other skin disorders, there are fewer racial differences, 3) there are many nail palates (20) in the limbs, and 4) it is easy to unify the composition of nail photographs to the square. In our analysis, we used R-CNN to automatically identify and extract part of the nail from individual clinical images.[8] Using the resulting dataset, we then trained CNNs to determine the accuracy of the binary classification of the sample (i.e. onychomycosis or not).

## Materials and methods

We used clinical images obtained from four hospitals (Asan Medical Center institutional review board approval no. S2016-2209-0001; 2017–0087) to construct nail datasets (Table 1). Data on patient demographics and clinical images were collected via a retrospective chart review, and all data were fully anonymized before we accessed them. We created dataset “A” (Asan Medical Center) with 598,854 clinical images acquired from 2003 to 2016 and then used different methods to generate the A1 and A2 datasets. To obtain the A1 dataset, the R-CNN used the entire set of cases, whereas we obtained the A2 dataset using the general method for which a clinical diagnosis had been confirmed by chart review.

**Table 1:** Summary of image characteristics and available demographic information.

	Asan^c^		Inje^d^		Hallym^d^	Seoul^d^
	A1	A2	B1	B2	C	D
No. of images	49567	3741	100	194	125	939
Patient demographics						
No. of unique individuals	4557^a^	484	57	61	55	169
Age, mean (SD), y	40.85 (21.64)	45.61 (20.36)	47.89 (22.62)	53.78 (20.19)	38.57 (15.38)	50.83 (19.52)
Male, total (%)	45.1%	41.3%	43.9%	52.5%	52.7%	46.2%
Diagnosis						
;No. of images / total (%) among images						
Onychomycosis	6673 (13.5%)	1567 (41.9%)	50 (50.0%)	173 (89.2%)	57 (45.6%)	500(53.2%)
Nail dystrophy	13769 (27.8%)	1095 (29.3%)	50 (50.0%)	21 (10.8%)	68 (54.4%)	439(46.8%)
Onycholysis	2397 (4.8%)	319 (8.5%)				
Melanonychia	2290 (4.6%)	359 (9.6%)	-	-	-	-
Other nail disorders^b^	4439 (9.0%)	-	-	-	-	-
Normal	19999 (40.3%)	-	-	-	-	-

^a^Unique patient codes and individual data, including age and sex, were available for 84.4% of the A1 dataset (n = 41,813 images).

^b^Subungual hemorrhage, paronychia, subungual fibroma, ingrown nail, pincer nail, periungual wart, etc.

^c^Diagnosis was based on the image findings (A1 dataset), whereas clinical diagnosis was identified in the chart (A2 dataset). Fungal culture was performed in 64.7% (313 of 484 patients) of the A2 dataset, and in frequency of positive cultures, onychomycosis specimens accounted for 34.6% of positive cultures (118 of 341 specimens), and non-onychomycosis specimens accounted for 3.4% (5 of 149).

^d^The inclusion criteria for onychomycosis in the validation dataset were a positive KOH test or fungus culture result, or the successful treatment with antifungal drugs. The inclusion criteria for nail dystrophy were a negative KOH test or culture result, unresponsiveness to antifungal medication, or responsiveness to a triamcinolone intralesional injection. All the cases of B1 dataset were confirmed by fungal culture and all the cases of D dataset were confirmed by KOH test.

**Fig 1:**
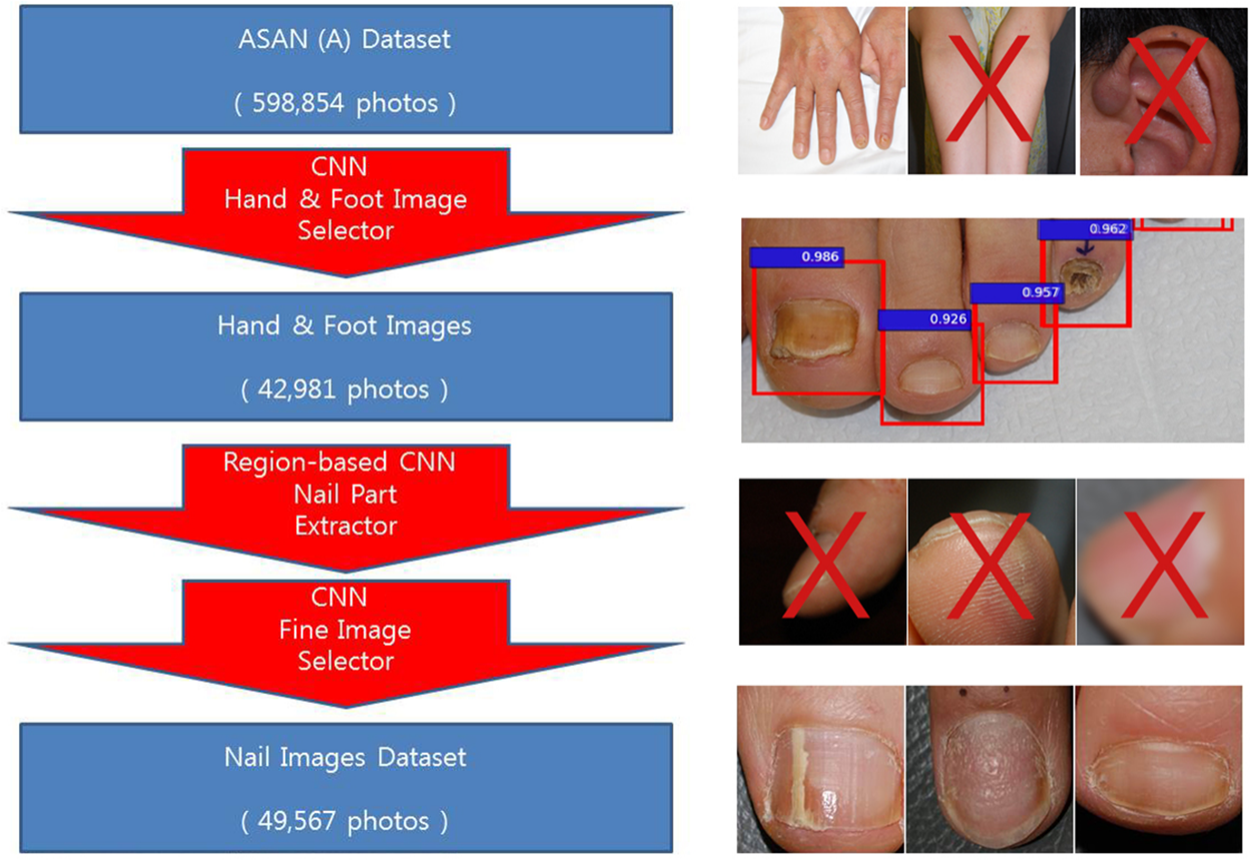
Automated establishment of the A1 dataset using R-CNN and two CNNs. 1) CNN hand & foot image selector which automatically detects and selects hand and foot images from the entire dataset. 2) R-CNN nail part extractor which identifies and crops part of the nail from individual clinical images by differentiating the nail plate from the background. 3) CNN fine image selector which rules out photographs with inadequate composition or focus.

We used one R-CNN (faster R-CNN, https://github.com/rbgirshick/py-faster-rcnn, model = VGG-16) and two CNNs (hand and foot image selector and fine image selector; CNN model = ResNet-152) to obtain the A1 dataset (Fig 1). Briefly, by first using a hand and foot image selector, we extracted 42,981 hand and foot photographs from 598,854 clinical photographs. Part of the nail in these images was then automatically cropped by the R-CNN trained with nail photographs.^3^ The R-CNN model had been trained using information about the crop location on the nail from 3,741 images of the A2 dataset (which was created before the A1 dataset). One dermatologist cropped all of the images from the A2 dataset for the R-CNN training, and LabelImg (https://github.com/tzutalin/labelImg) was used as an annotation tool. Finally, fine image selector trained with respect to ineligible photographs was then used to exclude unfocused photographs and those with either incomplete or tilted views of the nail (Fig 1). Even through it is possible to crop the nail part with R-CNN while skipping the first step of picking up the hand and foot photographs, there were too many inaccurate nail-like images such as teeth when the R-CNN was run with the entire photographs. We created hand and foot selector by training ResNet-152 with clinical photographs with lesion tags (class #1 = hand and foot, class #2 = other body locations), and run with the entire photographs. Approximately 80% of the extracted images correctly contained the part of hand and foot.

**Fig 2:**
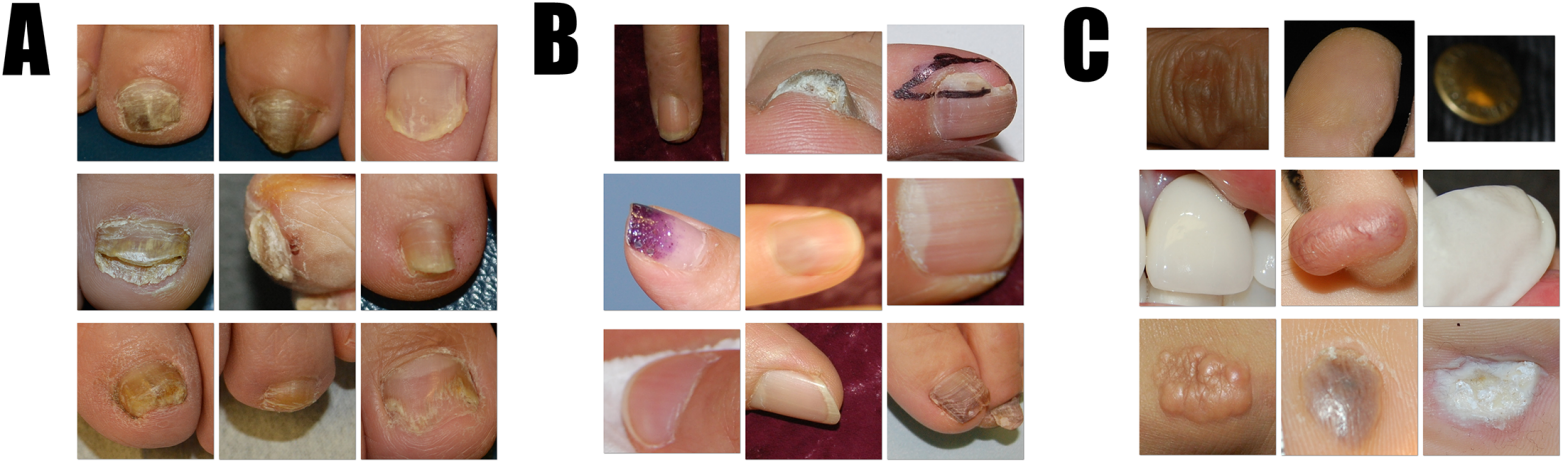
Examples of adequate, inadequate, and wrong images generated by R-CNN. (A) Adequate onychomycosis images; a nail plate occupies around 50~80% of the whole picture. (B) Inadequate nail plate images which include non-standardized nail images such as 1) a partially cut nail, 2) tilted nail, 3) a nail with a foreign substance, 4) unfocussed nail, 5) nail plates that are too small or too large to identify surrounding skin, and 6) image containing two or more nail plates. (C) Wrong images that included many general objects detected by R-CNN based on the difference between the nail and the background, but not corresponding to either. Objects that had a similar shape to a nail, such as teeth or warts were also generated. A large number of fingers and toes without a nail plate also came out.

Early in the process of selecting adequate images, manual classification was performed by dermatologists. At later stages however, the dermatologists supplemented the classifications initially made by the CNN (fine image selector). At first, we manually categorized all of the 2003 year images generated by the R-CNN as a wrong, inadequate, or adequate image, and trained the fine image selector with these images from 2003. The 2004 year images were next automatically classified by the fine image selector, and the misclassified images were manually corrected. We trained again the fine image selector using both the 2003 and 2004 year images to further improve accuracy. We repeated this process for all of the images from 2003 to 2016, after which the fine image selector had been improved to such an extent that only 1% of the images required manual correction. As a result, we retrieved 53,320 adequate nail images, 13,132 inadequate nail images, and 40,473 wrong images (Fig 2). Three dermatologists tagged the clinical diagnosis to the nail images generated by the R-CNN, with reference to the existing diagnosis tagged in the original image. For incoherent diagnosis cases, we reviewed the medical records to check for any alteration in the clinical diagnosis. If the diagnosis was still unclear, we dropped the image. When an original image was not tagged with a diagnosis, the three dermatologists tagged a diagnosis according to the image findings. Nail dystrophy comprises a mild ridge, pitting, and onychomadesis. We defined images as normal if we observed no change in the surrounding skin that also looked normal. If we observed any inflammatory patch or nodular lesion in the surrounding skin, we classified the corresponding images as “others.”

Among the 66,452 nail images obtained from the R-CNN, we excluded 13,132 inadequate images and 3,753 images of uncertain diagnosis to finally acquire 49,567 formalized nail photographs. We categorized these images into six classes as follows: 6,673 onychomycosis, 13,769 nail dystrophy, 2,397 onycholysis, 2,290 melanonychia, 19,999 normal, and 4,439 others (i.e., subungual hemorrhage, paronychia, subungual fibroma, ingrown nail, pincer nail, periungual wart, etc.).

We constructed the A2 dataset from 484 patients on the basis of the clinical diagnoses identified by chart review. We created the A1 and A2 datasets from the same pool of images.

The B, C, and D datasets (1,358 images) are validation datasets from three different sources (Table 1). The B1 dataset cases were confirmed by fungal culture and the D dataset cases by KOH test. The validation datasets and CNNs used in our current analyses are available at http://api.medicalphoto.org.

We created the E dataset to assess the performance of our automated onychomycosis database generation system by conducting a Web-based image search for “tinea”, “onychomycosis”, “nail dystrophy”, “onycholysis”, and “melanonychia” in English, Korean, and Japanese on http://google.com and http://bing.com, and downloaded a total of 15,844 images. From these images, the R-CNN created a nail dataset of 4,193 images. 861 images were automatically removed by fine image selector, and only 15 images manually excluded by a dermatologist. As a result, a total of 3,317 images were included in the E dataset. The CNN (model: ResNet-152 + VGG-19; arithmetic mean of both outputs; training dataset: A1) automatically classified the E dataset into six classes: 760 onychomycosis; 1,316 nail dystrophy; 363 onycholysis; 185 melanonychia; 424 normal; 269 others.

Using the Berkeley Vision and Learning Center deep-learning framework (BVLC) Caffe, we fine-tuned (in order to utilize the pre-trained earlier layers which contain more generic features, while maintaining some of the earlier layers relatively fixed, and updating some subsequent layers of the network) the ImageNet pretrained models of ResNet-152 (base_lr, 0.0001; max, 3 epochs; step, 2 epochs; gamma, 0.1; weight_decay, 0.00001; train_batch_size, 24) and VGG-19 (base_lr, 0.0001; max, 5 epochs; step, 5 epochs; gamma, 0.1; weight_decay, 0.0001; train_batch_size, 43).[9, 10] For data augmentation, we performed a 90-, 180-, and 270-degree rotation on the original images. All images were resized to 224x224 and a histogram normalization was performed as a preprocessing step before the training and testing of the models. We ran BVLC Caffe on Ubuntu 16.04 with an NVIDIA GTX 1070 (CUDA 8.0 and cuDNN 5.1).

After training the CNNs with four (onychomycosis, nail dystrophy, onycholysis, and melanonychia; A2 dataset) or six (onychomycosis, nail dystrophy, onycholysis, melanonychia, normal, and others; A1 dataset) classes, we tested them with two classes (onychomycosis or not).

**Fig 3:**
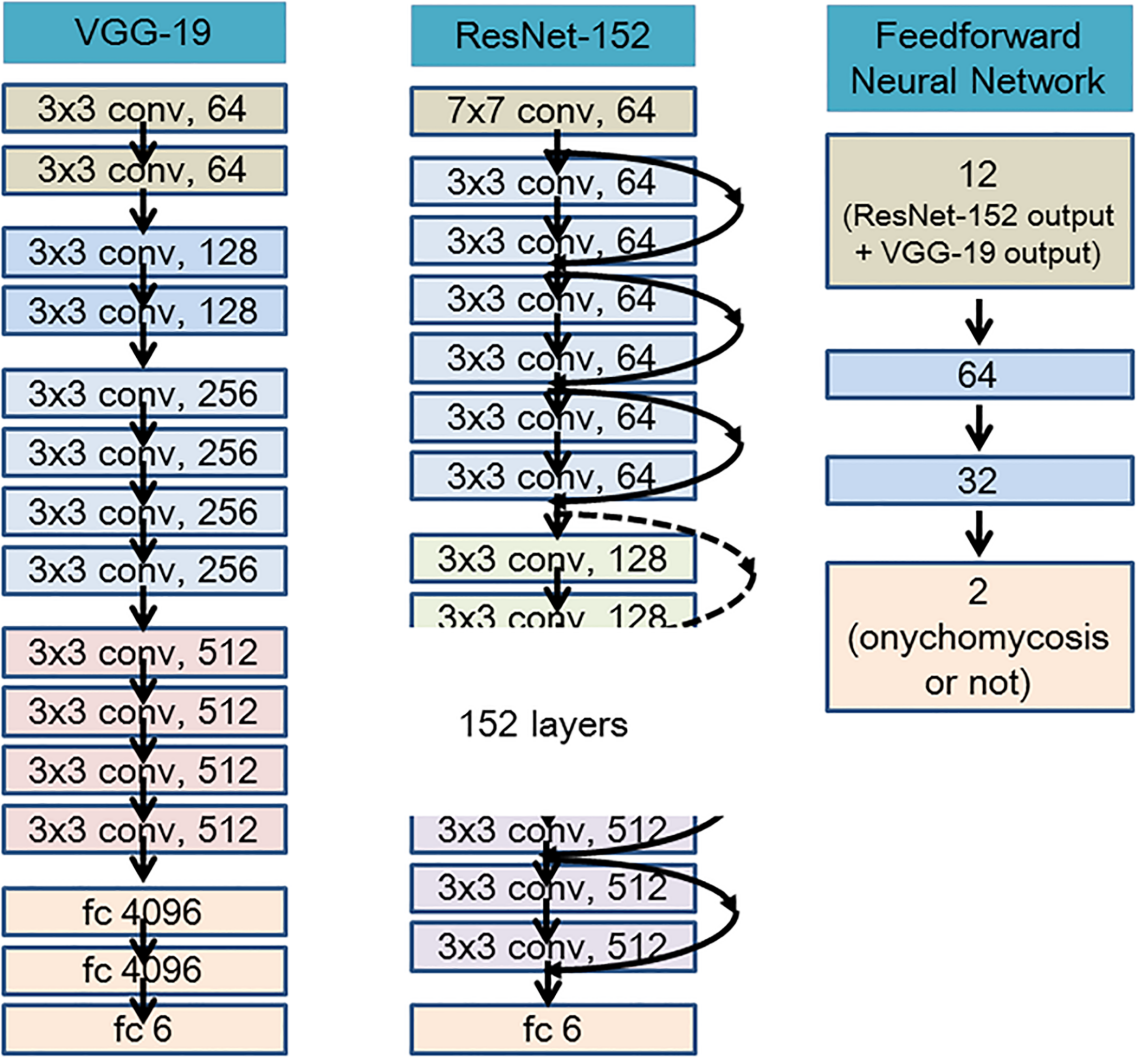
The representation of model architecture image for ResNet-152, VGG-19, and two-layered feedforward neural networks. 1) VGG-19 is a CNN model that have shown the importance of depth of the network in model performance. Using the simplest 3x3 convolution kernel throughout the whole network, VGG-19 won the ILSVRC in 2014. 2) ResNet-152 introduced the concept of residual learning in which the subtraction of feature is learned from the input of that layer by using shortcut connections (directly connecting input of (n)th layer to some (n+x)th layer, which is shown as curved arrow). It has proven that the residual learning can improve the performance of model training, especially when the model has deep network with more than 20 layers, and also revolve the problem of degrading accuracy in deep networks. 3) A total of 12 outputs from ResNet-152 and VGG-19 were used as input in a two-hidden-layered feedforward neural network. The final outputs (onychomycosis or not) were computed through the two hidden layers. ; fc is an abbreviation for fully connected. The numbers at each layer represent the number of nodes at each layer. ; Fig 3 was created based on the figure from the Ref #20. [20].

The final output was computed with two-layered feedforward neural networks to take advantage of ResNet-152 and VGG-19 with different characteristics (Fig 3). We refer to this as an “ensemble”, which combines the output of several models to produce a desired output. We used the ensemble method to get better result than the best-performing ResNet-152. When ResNet-152 sometimes made an error by predicting a wrong answer for a case that could have been easily diagnosed manually, we found that VGG-19 could then give the right answer, and vice versa. An ensemble of the outputs of ResNet-152 and VGG-19 can thus improve performance. For the sake of simplicity, we designated the “ensemble model” as the output of both the ResNet-152 and VGG-19 systems computed with the feedforward neural networks. Given that we had randomly set the initial values of the outer layer of the pretrained model when fine-tuning, we repeated the entire training process three times to adjust for possible deviations in the results, and then recorded the average value ± standard deviation of the output.

To identify differences in the performances of the A1 and A2 datasets, we trained ResNet-152 with each dataset and then performed the validation process using the B1, B2, C, and D datasets. Next, we used the A1 dataset to train the ensemble model to determine the best sensitivity and specificity of the AI system, which we also validated using the B1, B2, C, and D datasets. Lastly, in order to compare the diagnostic performances between AI, dermatologists, and the general population, we used the B1 and C datasets for validation. We used the B1 and C datasets to compare the performance of the CNNs with that of 42 dermatologists (16 professors, 18 clinicians with more than 10 years of experience in the department of Dermatology and 8 residents) and 57 individuals from the general populations (11 general practitioners, 13 medical students, 15 nurses in the dermatology department, 18 non-medical persons). We performed additional human tests using the B2 (194 images) and D (939 images) datasets for five dermatologists who had received the best grades in the Youden index in the previous test with the B1 and C test datasets. Images resized to 224x224 were used for the test by AI. In contrast, the human test was performed with the portable document format (pdf) of the images with an original resolution. Finally, we trained the ResNet-152 with the E dataset, a Web-based dataset created by CNNs (ResNet-152 + VGG-19; arithmetic mean of both outputs; training dataset: A1), and then validated its performance using the B1, B2, C, and D datasets. The diagnosis of these Web-based images is often uncertain, which makes it difficult to determine the classification performance of CNNs. The performance of the CNNs trained with the E dataset was indirectly estimated by the classification performance of the models with the B1, B2, C, and D validation datasets.

An additional experiment was implemented in order to assess the diagnostic accuracy of the models and the performance of fine image selector in assessing image quality with the change in the illumination and noise level of the images. The levels of brightness and noise were gradually changed to the 215 images from the B1 + C validation dataset. The ensemble model was used to measure area under the curve (AUC) from these 215 images and the average of adequate value from the fine image selector for these 215 images were recorded.

## Results

We obtained AUC results for receiver operating characteristic (ROC) curves and we describe the sensitivity/specificity values that maximize the sum of the sensitivity and specificity. When the VGG-19 was trained with the A1 dataset created by the R-CNN, the sensitivity/specificity/AUC results for the B1, B2, C, and D test datasets were as follows: (90.7 ± 3.1, 91.3 ± 1.2, 0.96 ± 0.02), (95.0 ± 2.0, 95.0 ± 0.0, 0.98 ± 0.02), (80.0 ± 2.0, 86.0 ± 6.2, 0.90 ± 0.06), and (87.3 ± 2.1, 68.0 ± 2.1, 0.82 ± 0.01), respectively. When the ResNet-152 was trained with the A1 dataset created by the R-CNN, the sensitivity/specificity/AUC results for the B1, B2, C, and D test datasets were: (94.7 ± 1.2, 98.0 ± 0.0, 0.98 ± 0.00), (90.3 ± 3.1, 95.0 ± 5.0, 0.97 ± 0.01), (86.7 ± 2.5, 79.0 ± 3.6, 0.90 ± 0.01), and (85.0 ± 2.0, 69.3 ± 0.6, 0.81 ± 0.00), respectively. These data indicated that we achieved on par or slightly better results when training with the ResNet-152 model than with the VGG-19 model. When we trained ResNet-152 with the A2 dataset, the results were as follows for the B1, B2, C, and D test datasets: (88.0 ± 2.0, 94.0 ± 4.0, 0.94 ± 0.01), (90.3 ± 6.7, 96.7 ± 5.8, 0.97 ± 0.01), (84.7 ± 8.7, 74.7 ± 6.7, 0.85 ± 0.00), and (88.0 ± 3.6, 56.0 ± 5.3, 0.76 ± 0.01), respectively. We achieved much better results when training with the A1 dataset than with the A2 dataset.

When the ensemble model was used with the A1 training dataset, the sensitivity/specificity/AUC results for the B1, B2, C, and D test datasets were: (96.0 ± 0.0, 94.7 ± 2.3, 0.98 ± 0.00), (82.7 ± 4.2, 96.7 ± 2.9, 0.95 ± 0.00), (92.3 ± 2.9, 79.3 ± 4.0, 0.93 ± 0.01), and (87.7 ± 4.0, 69.3 ± 4.9, 0.82 ± 0.00), respectively. When using the ensemble model, the AUC improved by 2.6% and 1.1% for the C and D test datasets, respectively, compared with the ResNet-152 model alone. The ensemble model also improved the AUC by 2.6% for the C test dataset compared with VGG-19 model alone.

**Fig 4:**
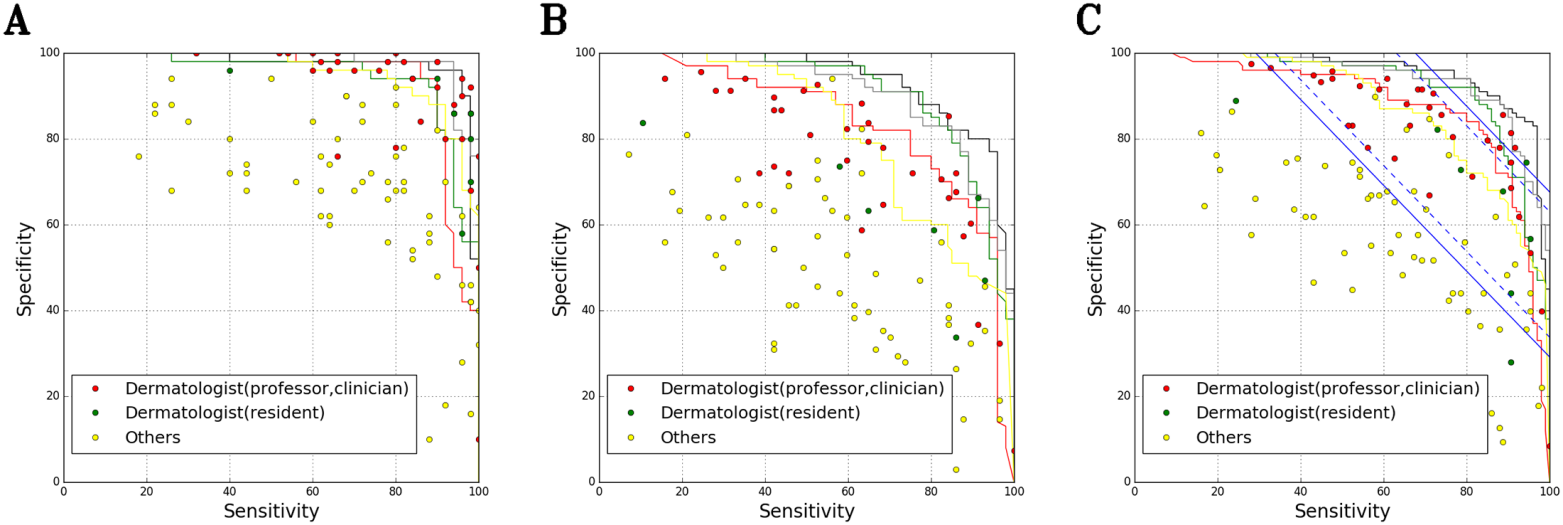
Evaluation of sensitivity/specificity in diagnosing onychomycosis and nail dystrophy for the B1 and C test datasets. (A) B1 test dataset: 100 images (50 onychomycosis and 50 nail dystrophy) (B) C test dataset: 125 images (57 onychomycosis and 68 nail dystrophy) (C) B1 + C test dataset: 225 images (107 onychomycosis and 118 nail dystrophy) Black curve: Ensemble model (ResNet-152 + VGG-19 + feedforward neural networks) trained with the A1 dataset; Gray curve: ResNet-152 trained with the A1 dataset; Red curve: ResNet-152 trained with the A2 dataset; Green curve: VGG-19 trained with the A1 dataset; Yellow curve: ResNet-152 trained with the E dataset; Red dot: 34 dermatologists (16 professors and 18 clinicians); Green dot: 8 dermatologists (8 residents); Yellow dot: 11 general practitioners, 13 medical students, 15 nurses in the dermatology department, and 18 non-medical personnel; Blue line: Youden index (sensitivity + specificity − 1) of 42 dermatologists with 99% confidence interval (CI); Blue dotted line: Youden index of 42 dermatologists with a 95% CI.

In the specificity/sensitivity graph obtained from the B1 and C test datasets, the performance of the CNN (ensemble model; training dataset = A1) surpassed that of almost all 42 dermatologists who manually processed the same samples. The data showed that 0.7 ± 0.6 and 6.0 ± 1.7 dermatologists performed better than the CNN with respect to the B1 and C datasets, respectively. When we combined both the B1 and C datasets, only one dermatologist performed as well or better than the CNN, but this was only once in three experiments (Fig 4)

The mean value of the Youden index (sensitivity + specificity − 1) of the 42 dermatologists was 48.39% (confidence interval (CI) (*p* = 0.01) 29.16–67.62%, CI (*p* = 0.05) 33.76–63.03%) when we combine the B1 and C dataset. When we conducted CNNs (ensemble model or ResNet-152) training with the A1 dataset, the AI Youden index was more than 67.62%, which is the upper limit for the 99% CI of dermatologists in most ranges. When we conducted CNN (ResNet-152) training with the A2 dataset, the Youden index was more than 63.03%, which is the upper limit for the 95% CI of dermatologists in most ranges (Fig 4C).

**Fig 5:**
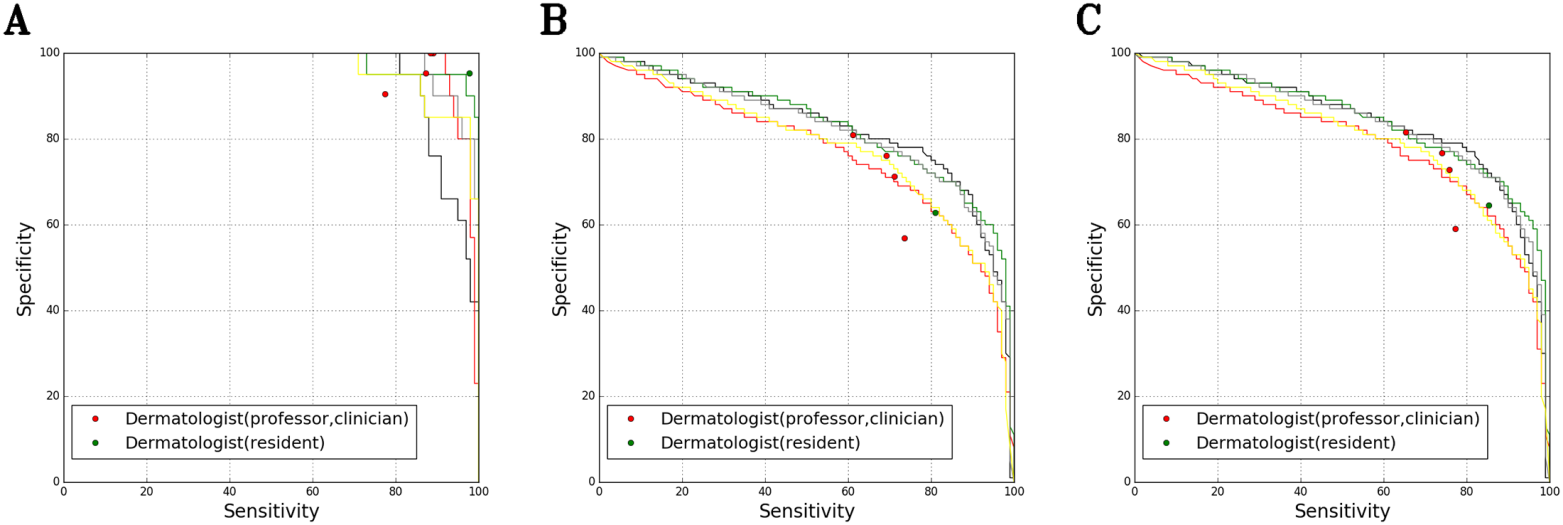
Evaluation of sensitivity/specificity in diagnosing onychomycosis and nail dystrophy for the B2 and D test datasets. (A) B2 test dataset: 194 images (173 onychomycosis and 21 nail dystrophy) (B) D test dataset: 939 images (500 onychomycosis and 439 nail dystrophy) (C) B2 + D test dataset: 1133 images (673 onychomycosis and 460 nail dystrophy) Black curve: Ensemble model (ResNet-152 + VGG-19 + feedforward neural networks) trained with the A1 dataset; Gray curve: ResNet-152 trained with the A1 dataset; Red curve: ResNet-152 trained with the A2 dataset; Green curve: VGG-19 trained with the A1 dataset; Yellow curve: ResNet-152 trained with the E dataset.

We obtained similar results from further validations with the B2 + D dataset (1,134 images) of the five dermatologists who performed best on the B1 + C dataset. Only one dermatologist performed better than the ensemble model trained with the A1 dataset, and only once in three experiments (Fig 5).

**Fig 6:**
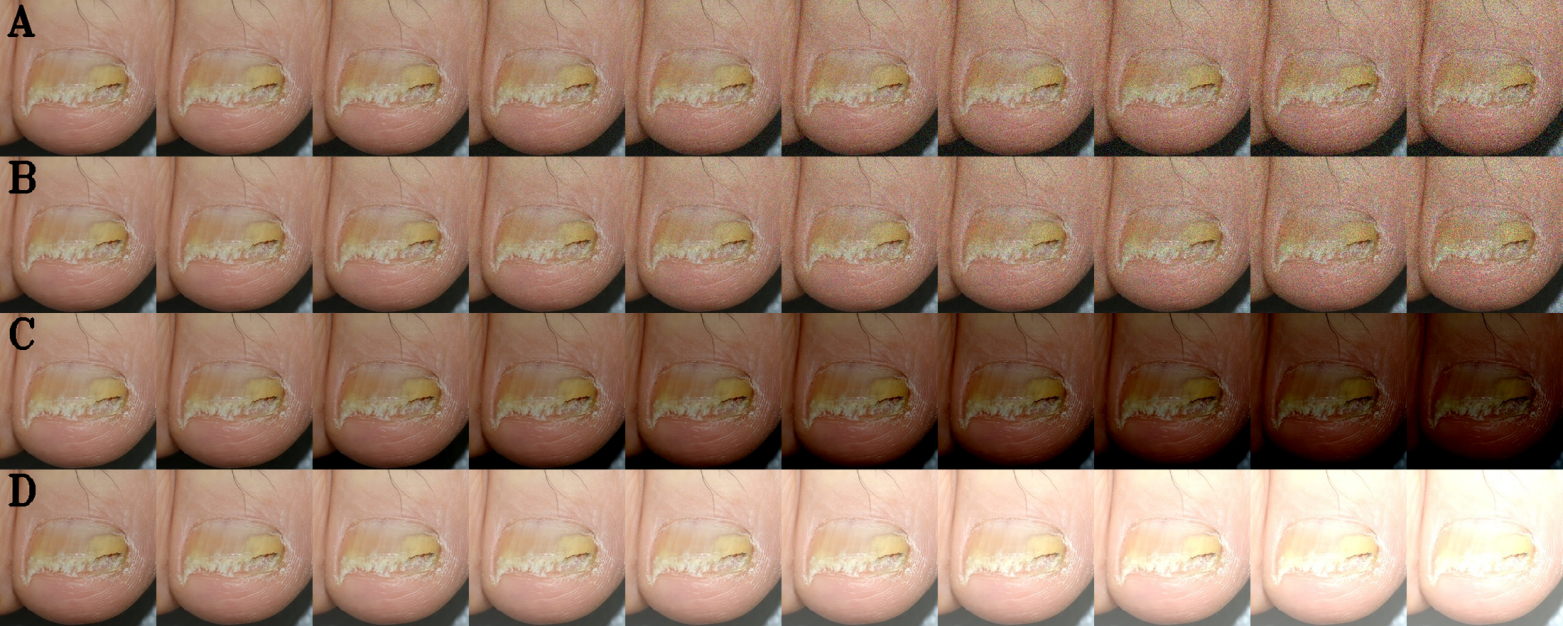
An example of nail images with the adjusted brightness and noise in 10 levels. (A) Noise (Gaussian); (B) Noise (Speckle); (C) Brightness (Black); (D) Brightness (White).

**Fig 7:**
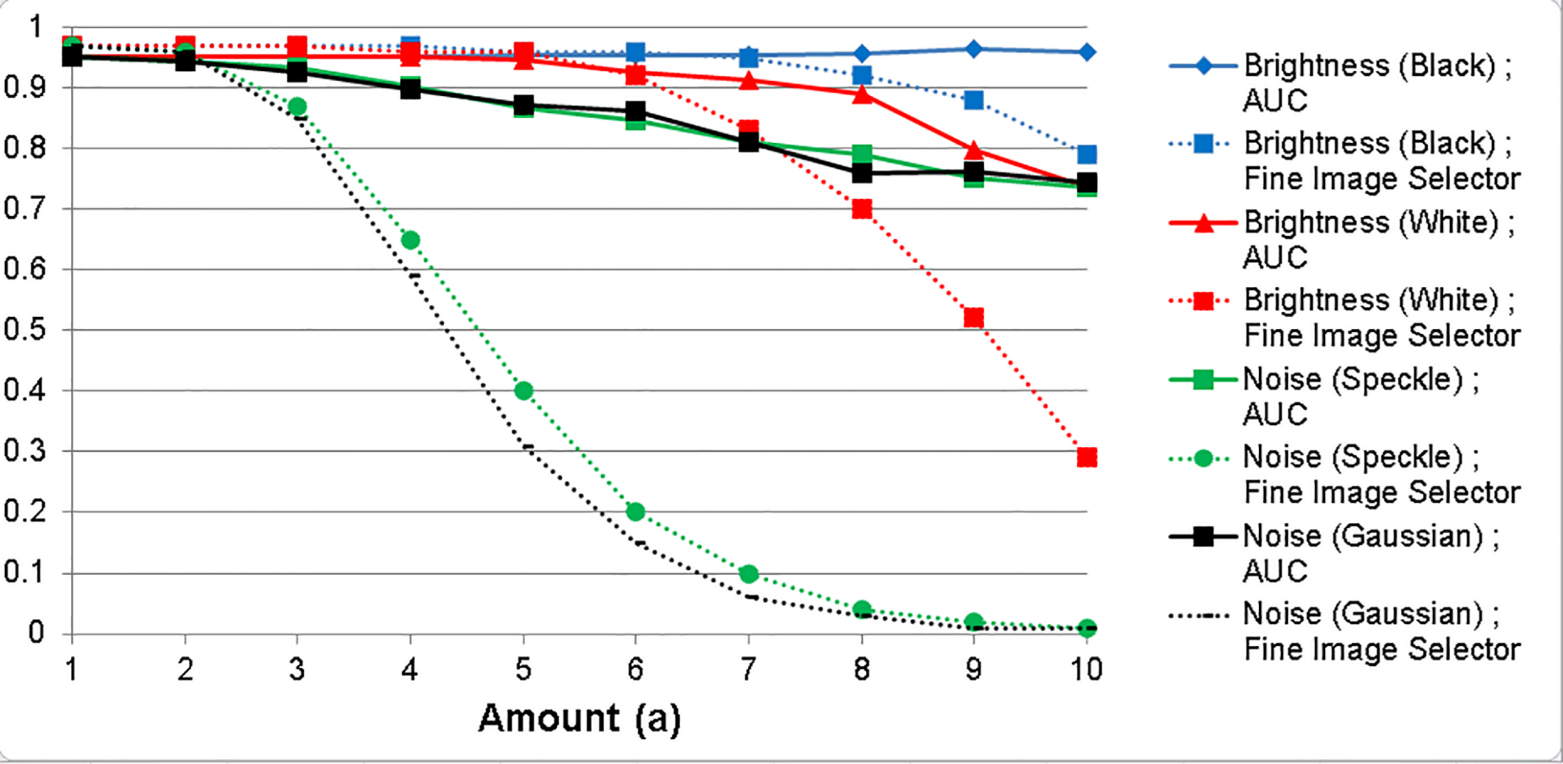
The change in the AUC of onychomycosis classification (ensemble model; test = B1 + C dataset) and the adequate value of the fine image selector. Fine image selector is a CNN model (ResNet-152) trained to analyze photos, categorize into adequate, inadequate, or wrong image, and produce an adequate value as an output. As the amount of added brightness and noise increased, the AUC (Test = B1 and C dataset), which represents the classification accuracy, and the adequate value of the fine image selector decreased.

Fine image selector is a CNN model (ResNet-152) trained with adequate, inadequate, and wrong images obtained from the building of A1 dataset. It outputs an adequate value based on the following criteria: 1) whether an image contains nail or not, 2) whether the composition of the nail image is appropriate, 3) whether the general image quality, such as focus, brightness, and noise, are appropriate. Figs 6 and 7 illustrates the results from the analysis with the change in the levels of brightness and noise. As you can see from the result, the AUC and the adequate value of the fine image selector was reduced with an increasing addition of the levels of brightness and noise. The adequate value of the fine image selector decreased much faster than the AUC with an addition of small levels of brightness and noise. The change in the level of brightness appeared to have smaller impact on the AUC than the change in the level of noise (Fig 7), which may be due to the histogram normalization and the consequent correction of the image brightness before the analysis.

## Discussion

Computer vision and object recognition technologies have made tremendous advances in recent years. Microsoft ResNet was reported to surpass human-level performance (5.1%) on visual recognition challenges.[5, 10] A deep-learning algorithm has also been reported to have achieved accuracy in skin cancer and diabetic retinopathy diagnoses that is comparable to those of medical experts.[1, 2] A few dermatologic studies have reported on the use of deep learning or machine learning.[2, 11–13] Recently, Esteva et al. demonstrated dermatologist-level classification of skin cancer by deep neural networks.[2] These researchers used 129,450 images to train the model into 757 classes, and ultimately validated the system using 2 classes (i.e. malignant or not).

An immense dataset is necessary however for deep learning to function effectively. For example, to perform the relatively simple task of distinguishing cats from dogs with 95% accuracy using deep learning, more than 1,000 photographs of each species are required. Considering the complexity of the task in diagnosing cancer many more medical photographs may be required for accurate medical diagnoses and retrieving sufficient numbers of such images may be difficult or even impossible in practical terms.

There are additional obstacles to the use of deep learning with photographs of human skin. Unlike fundoscopy images in ophthalmology and X-ray images, the clinical photographs used in dermatology are not standardized in terms of image composition. In addition, more than one skin lesion may be present on a single image. Images of nails tagged with onychomycosis may also contain other nails that are clinically normal or that have a different diagnosis such as nail dystrophy. This is a common problem with clinical dermatology images, and if the lesion is not extracted in the image, incorrect information that is irrelevant to the lesion may be transmitted.

Only low-resolution images can be used currently for training with deep-learning tools. Because of the limitations in graphics processing unit (GPU) memory, a 50,176 (224 × 224) to 89,401 (299 × 299) pixel-size is the highest resolution for an image that can be used in training. However, smartphones today are equipped with cameras with a resolution of >10,000,000 pixels. In fact, merely resizing clinical images without preprocessing would cause difficulties in image analysis, especially if the lesion of interest occupies only a small portion of the image.

R-CNN detects the location of lesions of interest by distinguishing them from the background.[7] The “faster R-CNN” system used in our current study is a type of R-CNN which can detect objects on a real-time basis by minimizing the time required for region proposal computation through the use of region proposal networks.[8] With “faster R-CNN”, we can quickly distinguish and extract the part of lesion or structure with a formalized composition from the entire dataset. Indeed, in our present analysis, we used “faster R-CNN” to acquire a sufficient number of adequately cropped standardized images of the nail with acceptable resolutions.

In comparing the CNNs trained with the A1 and A2 datasets, we obtained better results with the A1 dataset generated by R-CNN. Although some parts of the images of the A1 dataset were tagged according to the medical records, many were tagged according to the findings of three dermatologists. However, we obtained 49,567 high-quality images, which yielded a better result than those from CNNs trained with the A2 dataset composed of cases confirmed by chart review. An additional advantage of the R-CNN was that it could generate a training dataset with a distribution similar to the typical prevalence, thereby preventing selection bias in the training dataset and an erroneously skewed model output. Photographs of normal nails or mild nail dystrophy are difficult to find in hospital archives. With the help of R-CNN however, we obtained 19,999 images of normal nails and many of mild nail dystrophy, thereby enhancing the model’s diagnostic accuracy with respect to normal nails.

There have been no previous reports on the fast generation of large skin-disease datasets using R-CNN, which can also be applied to both the nodular and papular lesions of other skin disorders. For instance, R-CNN trained with nevi image datasets can be applied to the study of melanoma. Moreover, there are no data available for predicting the number of images required for deep learning in the field of dermatology. We used the A1 and A2 datasets as training datasets to perform a binary classification of ‘onychomycosis or not’. The A2 dataset, comprising 3,741 accurately tagged images (including 1,567 onychomycosis images), yielded successful results with a diagnostic accuracy comparable to that of the participant dermatologists. Even the E dataset, comprising 3,317 (720 onychomycosis images) relatively inaccurate tagged images, also produced a good result. Although additional studies are needed to confirm the number of images required to perform deep learning, we consider, based on our present observations, that approximately 1,000 standardized images per class or target disorder will be necessary to achieve good diagnostic accuracy for binary classification.

The tests previously used to establish the diagnosis of onychomycosis include direct potassium hydroxide (KOH) examination, culture, and nail clipping with Periodic acid-Schiff (PAS) staining, but none alone can be considered to be a standard test.[14] Sensitivity values have been reported at between 44% and 100% for the KOH test, at between 23% and 84.6% for culture, and between 81% and 91.6% for biopsy.[15–19] Because we can adjust the AI threshold for diagnosing onychomycosis, we can increase either the sensitivity or specificity for detecting onychomycosis depending on the threshold.

Confirmatory onychomycosis testing is recommended before systemic therapy. Nonetheless, there are circumstances in which such tests cannot be done, especially by general practitioners. However, with the advent of using smartphones to diagnose onychomycosis, it is expected that much medical and social benefit can be obtained through the enhancement of diagnostic accuracy in this way. We have created a website (http://nail.medicalphoto.org) for mobile devices to demonstrate the potential of using smartphones for this purpose. It must be noted that onychomycosis is not diagnosed using only one image in the clinic, but with consideration of many factors including the condition of the sole and other toenails as well as the results of other test methods. Hence, our current deep learning algorithm is expected to provide a supportive role in the diagnosis of onychomycosis.

The results from this study suggest that the CNNs developed in this study and the smartphone platform we developed may be useful in a telemedicine environment where the access to dermatologists is unavailable. When pictures with inadequate brightness or noise levels are supplied in such environment, the diagnostic accuracy may be affected. The fine image selector trained with inadequate images in this study may be used to send a cautionary warning to the user so that new photos with an adequate image quality are requested (Figs 6 and 7).

## Conclusions

Our here proposed R-CNN approach can be used to build a desired lesion dataset from existing photographs, and the accuracy of CNNs can be increased by increasing the number of images in the dataset. We conducted CNN training with datasets labeled according to the diagnosis of three dermatologists, based mainly on their assessment of images. This precisely matched the test datasets from three different sources. In diagnosing onychomycosis, CNNs trained with 49,567 photographs demonstrate higher diagnostic accuracy than dermatologists who participated in this study.
